# Vertical tunneling FET with Ge/Si doping-less heterojunction, a high-performance switch for digital applications

**DOI:** 10.1038/s41598-023-44096-5

**Published:** 2023-10-05

**Authors:** Iman Chahardah Cherik, Saeed Mohammadi, Subir Kumar Maity

**Affiliations:** 1 Department of Electrical and Computer Engineering, Semnan University, Semnan, 3513119111 Iran; 2 School of Electronics Engineering, Kalinga Institute of Industrial Technology (KIIT), Bhubaneswar, Odisha 751024 India

**Keywords:** Electrical and electronic engineering, Nanoscale devices

## Abstract

A vertical tunneling field effect transistor composed of a doping-less tunneling heterojunction and an n^+^-drain is presented in this paper. Two highly-doped p^+^ silicon layers are devised to induce holes in an intrinsic source region. Due to employing a double gate configuration and Hafnium in the gate oxide, our proposed structure has an optimized electrostatic control over the channel. We have performed all the numerical simulations using Silvaco ATLAS, calibrated to the verified data of a device with the similar working principle. The impact of the wide range of non-idealities, such as trap-assisted tunneling, interface trap charges, and ambipolar conduction, is thoroughly investigated. We have also evaluated the impact of negative capacitance material to further improve our device switching characteristics. Introducing both n-channel and p-channel devices, and employing them into a 6T SRAM circuit, we have investigated its performance in terms of parameters like read and write SNM. The FOMs such as *I*_*on*_ = 34.4 µA/µm, *I*_*on*_*/I*_*off*_ = 7.17 × 10^7^, and *f*_T_ = 123 GHz show that our proposed device is a notable candidate for both DC and RF applications.

## Introduction

Although conventional transistors with over barrier conduction mechanism are still the primary choice of nanoelectronics industry giants, the search for novel electronic devices with a different conduction mechanism and superior performance is continuously ongoing^1–3^. Tunneling field effect transistors (TFETs) are among most promising and successful alternatives^4,5^. They filter the high (low) energy electrons (holes) from the flowing charge carriers and form a tunneling energy window, theoretically leading to a very low off-state current^6^. The other advantage of such a filtering is that the subthreshold swing of tunneling FETs can beat the physically-limited subthreshold swing of MOSFETs. However, TFETs suffer from weaknesses such as low on-state current (*I*_on_)^7^ and trap-related problems^8^. The low on-current issue has been addressed in several studies and solutions have been suggested^9–21^. On the other hand, employing the charge plasma technique seems to be a viable solution for trap-related problems in TFETs. Kumar and Janardhanan proposed the first charge plasma based TFET in which to induce holes in the source region Platinum with the work function of 5.93 eV was used^22^. Doping-less TFETs (DL-TFETs) based on InGaN^23^ and heterojunction of GaAs_0.5_Sb_0.5_/In_0.53_Ga_0.47_As^24^ are the other proposed DL-TFETs, which benefited from the special features of III–V materials and achieved the on-state current of 80.2 µA/µm and 40.5 µA/µm, respectively. Sharma et al.^25^ suggested a DL-TFET with organic semiconductor, and obtained the *I*_on_ of 0.44 µA/µm and on/off current ratio (*I*_on_/*I*_off_) of 1.85 × 10^11^. Using negative capacitance dielectric materials^26^, 2D materials^27^, L-shaped gate^28^ and cladding layer concept^29,30^ are the other proposed solutions for improving TFETs performance.

In the conventional doping-less TFETs, inductive metals have been employed to induce charge carriers in the source and drain regions. Such a technique increases the risk of silicide formation. In this article, we propose a TFET which benefits from a doping-less tunneling junction. Two highly-doped silicon layers are advised to induce holes in the intrinsic source region, while using n^+^-doped silicon as the drain region can reduce fabrication steps and challenges. Removing inductive metal over the drain region and employing a heavily doped drain can also lessen the gate-to-drain capacitance, leading to better AC performance. Here, using germanium in the source region is preferred over silicon due to its smaller band gap and higher electron mobility^31,32^. Considering the scalability, choosing a vertical structure is better than a horizontal one.

## Device configuration, fabrication process, and simulation procedure

**Figure 1:**
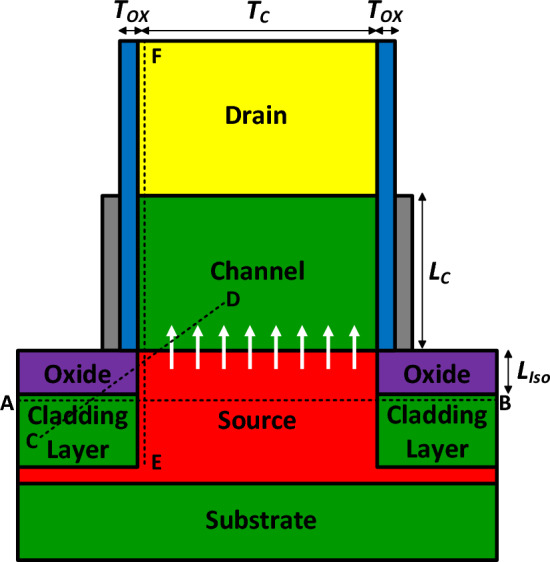
A schematic cross sectional view of vertical NCDL-TFET.

Figure 1 depicts our proposed vertical TFET. It includes two cladding layers to induce holes in an intrinsic Ge source region, while to reduce the fabrication process complexity an n^+^ Si drain region is deposited at the top of the structure. The material of the channel region is silicon, too. Because of employing the n^+^ drain and the cladding layer in our doping-less tunneling junction device, we name it NCDL-TFET. The electrostatic integrity of the gate is enhanced by using a 2 nm layer of Hafnium as the gate oxide. All other design parameters are tabulated in Table 1.

**Table 1:** Default values of the proposed TFET parameters.

Parameter	Value
Gate oxide thickness (*T*_*ox*_)	2 nm
Channel length (*L*_*C*_)	40 nm
Isolator length (*L*_*Iso*_)	3 nm
Channel thickness (*T*_*C*_)	10 nm
Source/channel doping (*N*_*SC*_)	1 × 10^15^ cm^−3^
Drain doping (*N*_*D*_)	3 × 10^18^ cm^−3^
Cladding layer doping (*N*_*Clad*_)	3 × 10^19^ cm^−3^
Gate metal work function (*WF*_*G*_)	4.38 eV

**Figure 2:**
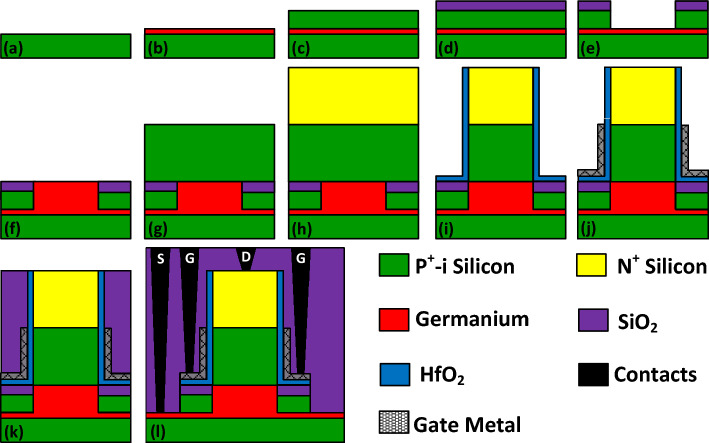
(**a**–**l**) Fabrication process steps of vertical NCDL-TFET structure.

Despite the complexity of fabrication process of nanoscale transistors, we suggest a fabrication process flow for realizing NCDL-TFET that, according to the literature^33,34^, practically seems feasible. At the beginning a germanium layer is grown over a silicon substrate (see Fig. 2a,b). With the low-temperature in-situ doping technique, a p^+^ silicon layer, acting as a p-gate, is deposited over the germanium layer (see Fig. 2c). Then a thin isolator layer of SiO_2_ is deposited over the silicon layer, following which a U-shaped trench is carved in the cladding layer (see Fig. 2d,e). In the next step, an intrinsic germanium layer is grown in this trench to act as the source region (see Fig. 2f). The layers of intrinsic and n^+^-doped silicon are deposited to form channel and drain regions, respectively (see Fig. 2g,h). After selective etching of the two above mentioned layers, the high-k gate oxide is deposited (see Fig. 2i). The gate metal is deposited, followed by the deposition of the SiO_2_ as the spacer (see Fig. 2j,k). Finally, the source, gate, and drain contacts are connected (see Fig. 2l).

**Figure 3:**
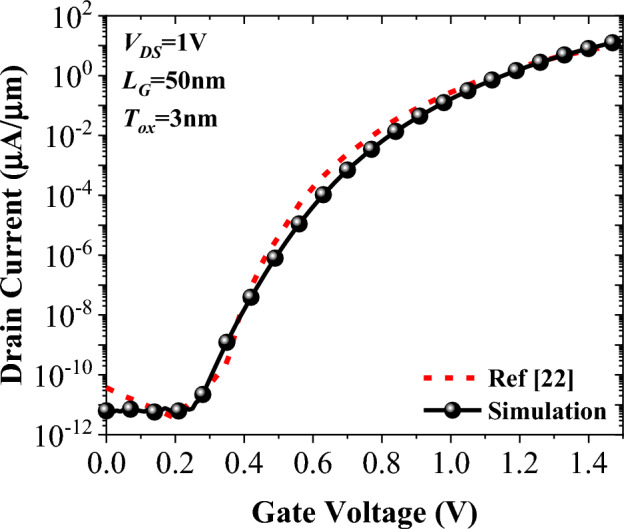
Comparison between the measured transfer characteristic of the doping-less TFET^22^ and the reproduced curve by our calibrated simulation setup.

In Fig. 3, we have drawn the extracted *I*_D_–*V*_G_ curve of Ref.^22^ alongside the reproduced result of our device simulator, and it can be inferred that a good matching is obtained. Silvaco ATLAS device simulator was employed to assess our device performance^35^. We have used Shockley–Read–Hall (SRH) recombination model, CVT, fermi, and dynamic non-local band-to-band-tunneling (BTBT) models for all the simulations. The charge transport model of drift–diffusion is also activated. Due to the large indirect band gap of silicon and channel thickness of 10 nm quantum confinement model is not incorporated in the simulations. During the calibration process, m_e_ = 0.22m_0_ and m_h_ = 0.17m_0_ have been utilized.

## Simulation results

This section first evaluates the impact of cladding layers on the DC/RF performance of NCDL-TFET. Then, we assess our device performance in the presence of trap-assisted tunneling (TAT) and interface trap charges (ITC), temperature change, and ambipolar conduction. Then, we incorporate a negative capacitance in the design of NCDL-TFET, and its impact on our device performance is investigated. Finally, by designing a p-type doping-less TFET, a 6T SRAM cell is designed and its performance in terms of parameters such as reading and writing static noise margin (SNM) is evaluated.

**Figure 4:**
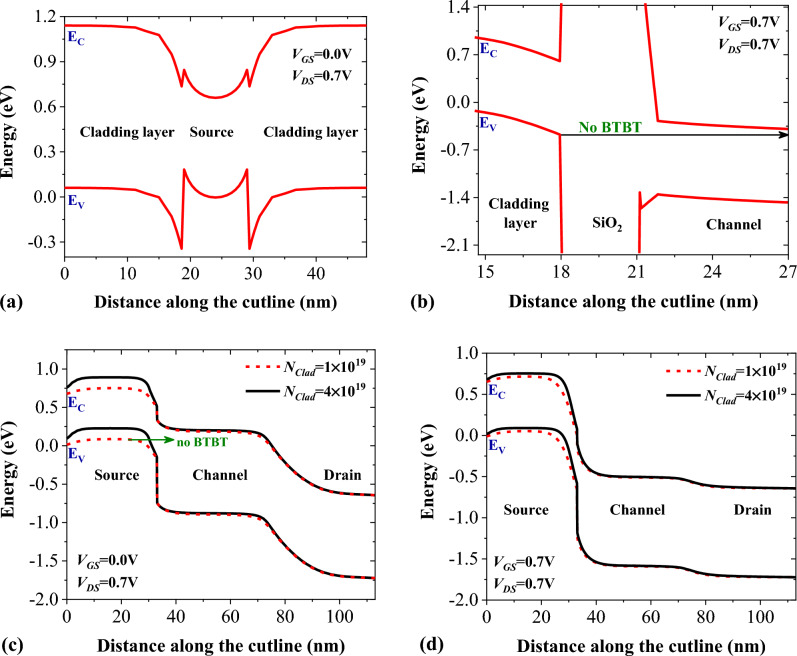
The energy bands diagram of NCDL-TFET along (**a**) A-B cutline and (**b**) C–D cutline. Impact of cladding layers doping concentration on the energy bands diagram of NCDL-TFET along the E–F cutline in (**c**) off-state and (**d**) on-state.

Energy bands diagrams of NCDL-TFET along the A-B and C-D cutlines are depicted in Fig. 4a and b. As it can be inferred from Fig. 4a, no charge transport occurs between the cladding layers and the source region, mainly because of a large valence band offset between the mentioned regions. Figure 4b also depicts that no band-to-band tunneling happens in the parasitic tunneling path, which is from the valance band of the cladding layers to the conduction band of the channel. Thermionic emission is also suppressed due to the large band gap of SiO_2_ and p^+^ doping of silicon layers. Figure 4c shows the energy bands profile of NCDL-TFET along the $\overline{\mathrm{EF} }$ segment (as displayed in Fig. 1) in the off-state (*V*_GS_ = 0 V) for two different doping levels of the cladding layer. It can be seen that the higher doping of the cladding layers contributes to a smaller barrier width and a more-abrupt tunneling junction at the source-channel interface. This is mainly because increasing cladding layer doping levels induces more holes in the intrinsic source region. Figure 4d illustrates the energy bands diagram in the on-state (*V*_GS_ = 0.7 V), indicating that a more-favorable tunneling junction is obtained by using higher level of doping concentration in the cladding layers.

**Figure 5:**
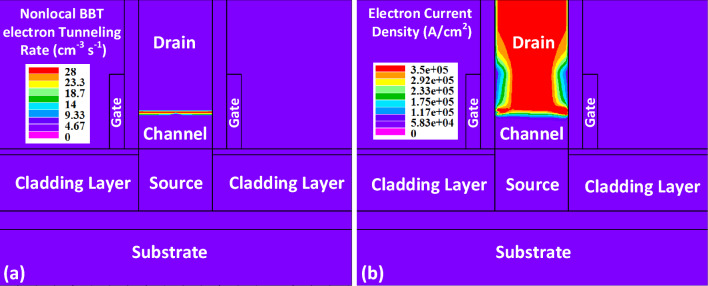
(**a**) Electron BTBT tunneling rate, and (**b**) electron current density contour maps at *V*_*GS*_ = 0.7 V and *V*_*DS*_ = 0.7 V.

According to Fig. 5a, the band-to-band tunneling rate has almost a horizontally uniform profile across the channel. Such a uniformity facilitates having a higher on-state current. The tunneling charge carriers move toward the drain side of NCDL-TFET, as represented in Fig. 5b.

**Figure 6:**
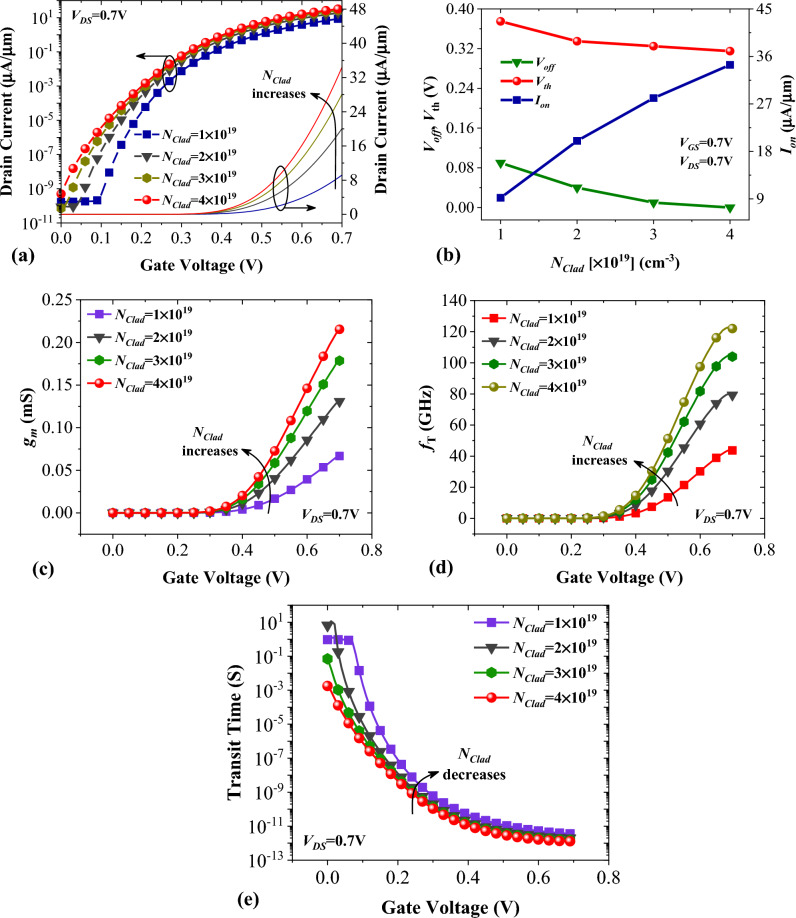
Impact of cladding layers doping concentration on (**a**) transfer characteristic, (**b**) *V*_*th*_, *V*_*off*_, and *I*_*on*_, (**c**) transconductance, (**d**) cut-off frequency and (**e**) transit time of vertical NCDL-TFET.

As discussed earlier, the doping density of cladding layers plays a significant role in the modulation of the energy bands profile at the source-channel junction of NCDL-TFET. In Fig. 6a, we have plotted the transfer characteristics of our device for four different values of *N*_*Clad*_. It can be seen that the device with *N*_*Clad*_ = 4 × 10^19^ cm^−3^ has a better performance compared with that of its counterparts. In Fig. 6b, the impact of cladding layers doping concentration on the *V*_*off*_, *V*_th_, and *I*_*on*_ is evaluated. We have defined *V*_*off*_ as the gate voltage in which BTBT is triggered. For the calculation of *V*_th_, we have employed the constant current method, as mentioned in^23^. Obviously, when *N*_*Clad*_ = 4 × 10^19^ cm^−3^ the device shows the best performance with minimum *V*_*off*_ and *V*_th_ and maximum *I*_*on*_ of 34.4 µA/µm. Transconductance, given by *g*_*m*_ = ∂*I*_*D*_/∂*V*_*GS*_, is another important parameter for evaluating FETs' performance. From Fig. 6c, it can be inferred that increasing *N*_*Clad*_ results in higher *g*_*m*_ values, which are desirable for high-performance devices in low-noise, high-frequency applications. Another essential RF performance metric of a FET device is cut-off frequency (*f*_*T*_). It is a function of transconductance and parasitic capacitances of the device. Figure 6d illustrates the impact of *N*_*Clad*_ on the *f*_*T*_ of NCDL-TFET. According to this figure, a significant frequency of 123 GHz can be achieved with *N*_*Clad*_ = 4 × 10^19^ illustrating that the role of higher *g*_*m*_ is more critical than increased parasitic capacitance. In Fig. 6e, we can see how the amount of *N*_*Clad*_ affects the transit time of charge carriers in NCDL-TFET. Transit time, which represents the time it takes for charge carriers to move from the source to the drain side, is inversely proportional to *f*_*T*_, where τ = 1⁄(2π) × *f*_*T*_^36^. The results show that when *N*_*Clad*_ is at 4 × 10^19^, the transit time values are the lowest, indicating that charge carriers require less time to move from the source to the drain side.

**Figure 7:**
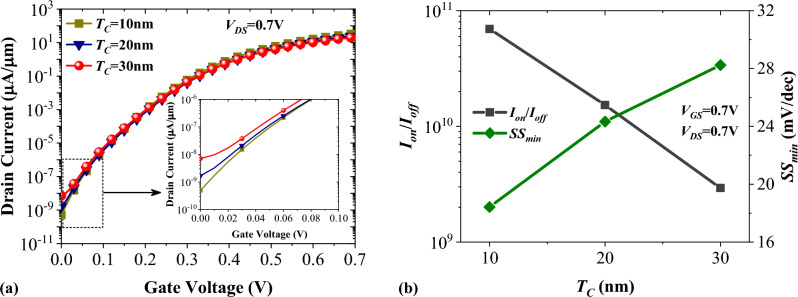
Impact of channel thickness on (**a**) transfer characteristic, and (**b**) *I*_*on*_/*I*_*off*_, and *SS*_*min*_ of vertical NCDL-TFET.

Figure 7a shows the impact of channel thickness on the transfer characteristics of NCDL-TFET. Increasing the channel thickness degrades the electrostatic control of the gate, contributing to higher off-state currents. We can see that tripling the channel thickness leads to an increment of *I*_*off*_ from 4.94 × 10^−10^ to 7.11 × 10^−9^ µA/µm. A slight reduction of on-state current with the increment of the channel thickness is also attributed to the electrostatic control degradation. However, the abovementioned values ensure us quantum confinement has no considerable impact on the on-state current of NCDL-TFET when *T*_*C*_ = 10 nm. In Fig. 7b, it is observable that with increasing *T*_*C*_ from 10 to 30 nm, the on/off ratio decreases from 6.96 × 10^10^ to 2.93 × 10^9^. Moreover, minimum subthreshold swing with ~ 153% increase reaches from 18.42 to 28.24 mV/dec.

**Figure 8:**
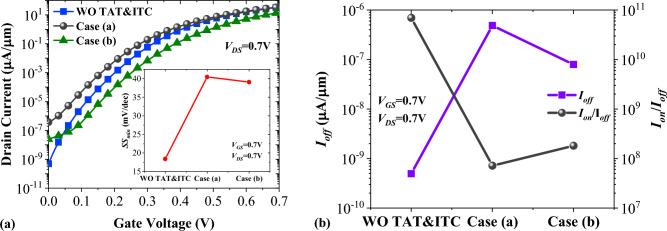
Impact of trap-assisted tunneling and interface trap charges on (**a**) transfer characteristic and (**b**) *I*_*off*_ and *I*_*on*_/*I*_*off*_ of vertical NCDL-TFET.

Defect at the heterojunctions and multi-phonon excitation at the oxide–semiconductor interface can adversely impact the performance of TFETs^8^. In Fig. 8a, we compare the impact of both non-idealities on the performance of our suggested device. In case (a), we have defined the trap energy (*E*_*t*_) of 0.2 eV and the trap density (*D*_*t*_) of 1 × 10^12^ cm^−3^ at the Ge-Si interface and the interface trap density (*D*_it_) of 3 × 10^12^ cm^−2^ eV^−1^ at the HfO_2_-Si interface^37^, while in case (b), a hetero-oxide interface (comprising of 0.5 nm SiO_2_ and 1.5 nm HfO_2_) has been employed. So, the *D*_*it*_ = 3 × 10^11^ cm^−2^ eV^−1^ was used in the INTERFACE model of the device simulator. The change in the subthreshold swing of NCDL-TFET at the presence of these detrimental effects is shown in the inset of the figure. Figure 8b illustrates that by including both TAT and ITC models *I*_*off*_ with almost three decades increment reaches to 4.88 × 10^−7^ µA/µm, leading to nearly three decades reduction of the on/off currents ratio from 6.96 × 10^10^ to 7.17 × 10^7^. On the other hand, employing a hetero-oxide interface improves the off-state current and the on/off currents ratio. However, it leads to a more complicated fabrication process.

**Figure 9:**
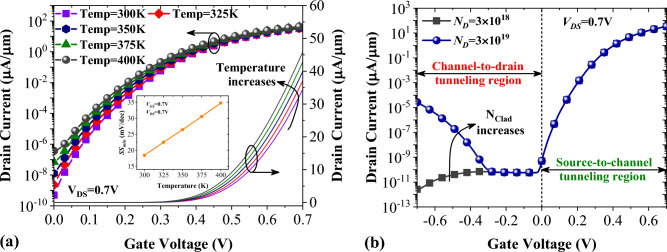
(**a**) Impact of temperature variation and (**b**) impact of drain doping concentration on the transfer characteristic of vertical NCDL-TFET.

The impact of temperature variation on our NCDL-TFET performance is investigated in Fig. 9a, where we have used temperatures ranging from 300 to 400 K. Since thermal generation of charge carriers plays a significant role in subthreshold conductance, the temperature's impact on the off-state current is more significant than that on the on-state current. However, the *I*_*off*_ = 3.65 × 10^−7^ µA/µm shows that NCDL-TFET has lower power dissipation than the short channel MOSFET even at temp = 400 K. In contrast, the on-state current remains almost unchanged, mainly because the transmission probability equation defined in Ref.^4^ has no direct dependency on the temperature. The inset of Fig. 9a shows that with an 100 K increment of temperature, the *SS*_min_ increases from 18.42 to 30.51 mV/dec which is still significantly lower than subthreshold swing of MOSFETs.

In order to suppress ambipolar conduction in the proposed device, the drain region of the device has not been heavily doped. As depicted in Fig. 9b, by choosing *N*_*D*_ = 3 × 10^18^ cm^−3^, the ambipolar current (*I*_*amb*_) is even lower than the off-state current. By one order of magnitude increase in *N*_*D*_ the ambipolarity of NCDL-TFET rises about seven decades. As a result, the *I*_*on*_/*I*_*amb*_ ratio reaches from 1.29 × 10^13^ to 1.25 × 10^6^, exhibiting dramatic reduction of our device reliability.

Comparing the achieved performance of our proposed device with some of the recently published works on the same topic, listed in Table 2, indicates that NCDL-TFET is a notable candidate for CMOS applications.

**Table 2:** Switching performance comparison of some recently introduced doping-less TFETs.

References	Material	*I*_*on*_ (µA/µm)	*SS*_*avg*_ (mV/dec)	*I*_*on*_/*I*_*off*_	*f*_*T*_ (GHz)	*V*_*Bias*_ (V)	*QC*	*ITC*	*TAT*
^22^	Si	11	~ 100	1.1 × 10^12^	–	1.5	✗	✗	✗
^23^	InGaN	80.2	7.9	8.02 × 10^13^	119	0.5	✗	✗	✓
^24^	GaAs_0.5_Sb_0.5_/In_0.53_Ga_0.47_As	40.5	20.3	4.86 × 10^9^	~ 75	0.6	✗	✓	✗
^25^	CH_3_NH_3_PbI_3_	0.44	27.33	1.85 × 10^11^	268	1	✓	✗	✓
^26^	Negative Cap-Si	~ 36	~ 65	~ 10^11^	–	1.2	✗	✗	✓
^27^	WTe_2_	222	54.15	2.22 × 10^5^	–	0.5	Atomistic simulation		
^28^	Si	10	40	~ 10^12^	4.5	1	✗	✗	✓
^29^	Ge-Si	27.28	42.43	8.24 × 10^8^	89.31	0.7	✗	✓	✓
		4.07	40.26	1.2 × 10^10^			✓	✗	✓
^30^	Ge-Si	7.57	18.65	1.48 × 10^8^	–	1	✓	✓	✓
This work	Ge-Si	34.4	51.78	7.17 × 10^7^	123	0.7	✗	✓	✓

**Figure 10:**
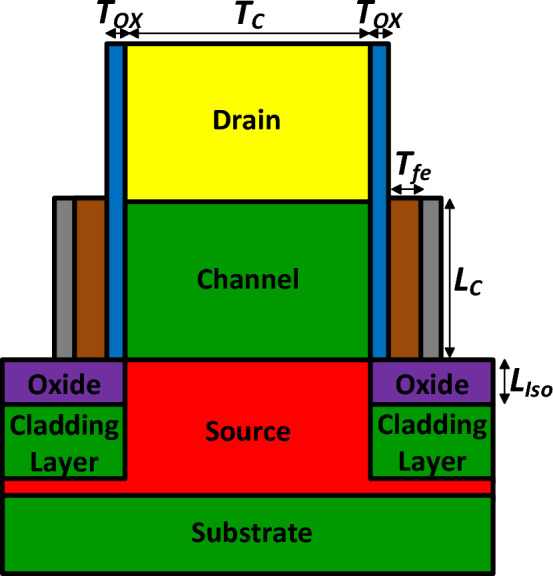
A schematic cross sectional view of a negative capacitance-based vertical NCDL-TFET.

**Figure 11:**
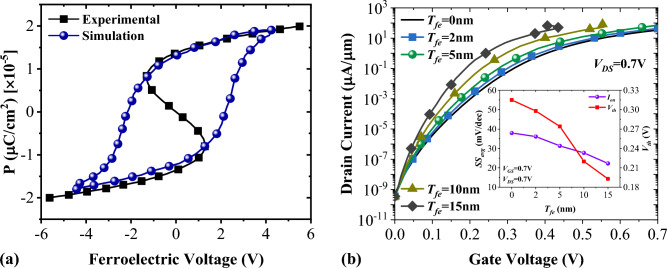
(**a**) Calibration of the p–v curve using experimental data from Ref.^41^ and (**b**) the impact of thickness of ferroelectric material on the transfer characteristics of NCDL-TFET.

Paraelectric Hafnium, a CMOS-compatible material, has a high potential to act as a negative capacitance material. Adding materials such as Si^38^, Al^39^, and ZrO_2_^40^ into paraelectric HfO_2_ can be a viable solution to attain ferroelectricity. We have employed yttrium-doped HfO_2_ (Y: HfO_2_) in a Metal-ferroelectric-insulator-semiconductor (MFIS) configuration to enhance our device switching performance, as illustrated in Fig. 10. To evaluate the impact of Y: HfO_2_ material on the performance of NCDL-TFET, we first calibrated our device simulator with the extracted polarization curve of^41^ (see Fig. 11a). Then, the on-state current is calculated using the procedure explained in our previous work^42^. Figure 11b shows the impact of the thickness of (Y: HfO_2_) on the *I*_*D*_-*V*_*G*_ curve of our proposed structure. It should be noted that although increasing *T*_*fe*_ improves the subthreshold swing, it can lead to more considerable hysteresis, which is not a desirable in CMOS applications. The inset shows the extracted values of *V*_th_ and *SS*_*avg*_, and it can be inferred that the higher values of *T*_*fe*_ lower *V*_th_ and improve *SS*_*avg*_.

**Figure 12:**
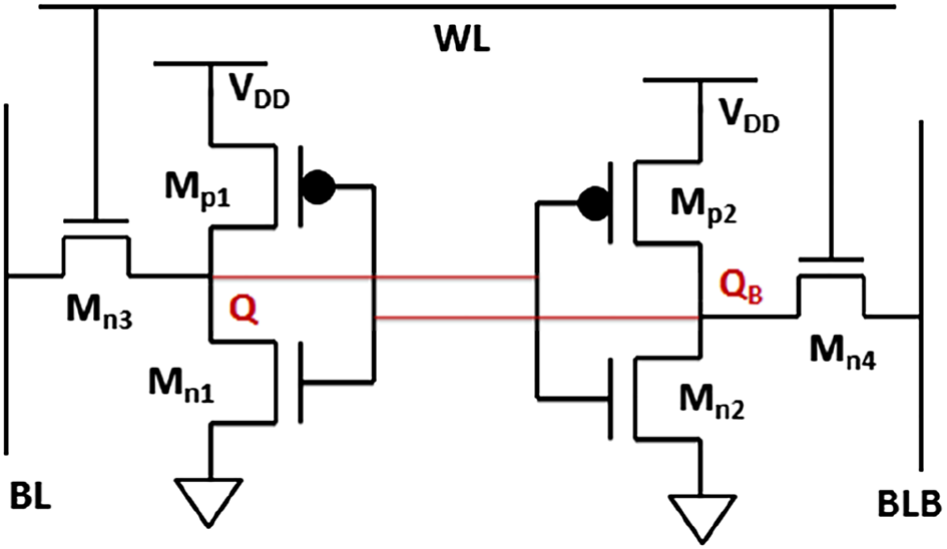
Schematic of a 6T SRAM cell.

**Figure 13:**
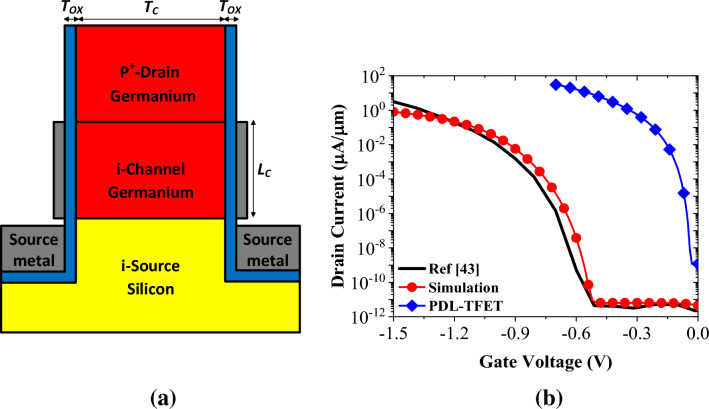
(**a**) A schematic cross sectional view of vertical PDL-TFET and (**b**) comparison between the measured transfer characteristic of the doping-less P-TFET of^43^ and the reproduced curve by our calibrated simulation setup. The transfer characteristic of vertical PDL-TFET is also plotted in the figure.

A more pragmatic assessment of a device's performance can be achieved by incorporating it into a familiar circuit configuration. In pursuit of this objective, our attention shifts to a hybrid six-transistor (6T) SRAM cell, depicted in Fig. 12. This configuration comprises four n-channel and two p-channel devices. The p-channel device chosen is a vertical PDL-TFET, designed along the same principles as the NCDL-TFET. According to the device structure exhibited in Fig. 13a, PDL-TFET has an intrinsic silicon source located at the bottom of the transistor, while germanium is employed in the channel and drain regions. All pertinent dimensions, parameter values, and activated models for simulations remain consistent with those utilized in NCDL-TFET simulations, with the exception of gate and source work functions which are assigned values of 4.65 eV and 3.9 eV, respectively. In Fig. 13b, our device simulator's accuracy is validated against simulation outcomes from the device highlighted in Ref.^43^. Additionally, we have plotted the transfer characteristics of PDL-TFET, which shows more-optimum performance for low-power applications.

**Figure 14:**
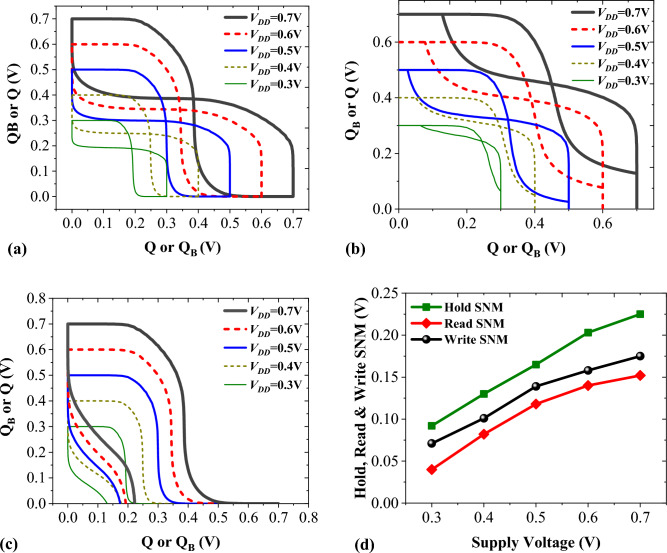
(**a**) Hold (**b**) Read and (**c**) Write SNM of 6T hybrid SRAM cell for different supply voltages. (**d**) Summary of Hold, Read and Write SNM for different supply voltages (*V*_*DD*_).

By incorporating both n-channel and p-channel devices, we construct the 6T hybrid SRAM cell using four NCDL-TFET devices (M_n1_, M_n2_, M_n3_, and M_n4_) along with two PDL-TFET devices (M_p1_ and M_p2_). The stability performance of the 6T hybrid SRAM cell is explored through the examination of different static noise margins (SNMs) and their alterations in response to varying supply voltages (V_DD_). To ascertain the SRAM cell's SNM, the voltage transfer characteristics (VTC) of the two cross-coupled inverters are plotted. Various SNM values can be derived as the cell functions in the HOLD, READ, and WRITE modes. When the cell is in a HOLD or data retention state, the word line (WL) remains inactive, ensuring that the access transistors remain off. In this state, the cell retains its data using the cross-coupled inverters. Figure 14a visually presents the properties of the HOLD SNM and their shifts corresponding to different supply voltages (V_DD_). Notably, the HOLD SNM diminishes proportionally as the supply voltage decreases^44^.

During the READ operation, the SRAM cell is highly susceptible to noise^45^. Following a READ action, the cell must preserve its state without erasing the stored value. Bit lines (BL and BLB) are prepared for the READ operation prior to activating the word line (WL). The READ SNM of the SRAM cell, observed across various supply voltages, is illustrated in Fig. 14b. The minimal bit line voltage required to alter the cell's state is referred to as the WRITE margin. During the WRITE operation, data bits are transmitted on BL and BLB before enabling the word line. The WRITE action's properties in terms of SNM are portrayed in Fig. 14c. A summary of the HOLD, READ, and WRITE noise margins for different supply voltages is presented in Fig. 14d. Across all scenarios, the noise margin decreases with diminishing supply voltage, rendering the SRAM cell increasingly unstable.

## Conclusion

We have suggested a TFET composed of a doping-less tunneling heterojunction and an n^+^-drain region in a vertical configuration. Due to using heterojunction of germanium/silicon in the tunneling interface, the on-state performance of our proposed device is more robust than Si-based doping-less TFETs. By employing a virtual fabrication process, we have shown that our structure can be realized in a feasible and convenient manner. Using Silvaco ATLAS, we have assessed the effect of non-idealities, such as defects at the tunneling interface and temperature, on our device performance. Our proposed device offers high scalability, sub-60 mV/dec performance even in the presence of defects, and higher AC performance than conventional doping-less TFETs. Although, using n^+^ doping in the drain region can increase the thermal budget but effectively reduce gate-to-drain parasitic capacitance and reduce fabrication steps. Moreover, silicide formation is no longer a challenge due to using highly doped cladding layers instead of inductive metal. We have also utilized yttrium-doped Hafnium as a negative capacitance material in an MFIS configuration, and a considerable improvement without any hysteresis was obtained. A CMOS-compatible p-TFET was also designed to meet all the requirements for developing a SRAM cell. The parameters such as *I*_*on*_ = 34.4 µA/µm, *SS*_*avg*_ = 51.78 mV/dec, and *f*_T_ = 123 GHz illustrate that NCDL-TFET is a notable candidate for high-performance applications such as designing SRAMs with lower power dissipation.

## Data Availability

The datasets used and/or analyzed during the current study available from the corresponding author on reasonable request.
